# Association of HIF-1α and NDRG2 Expression with EMT in Gastric Cancer Tissues

**DOI:** 10.1515/biol-2019-0025

**Published:** 2019-07-22

**Authors:** Ren-Xiang Wang, Xia-Wan Ou, Ma-Fei Kang, Zu-Ping Zhou

**Affiliations:** 1 Department of Medical Oncology, The Affiliated Hospital of Guilin Medical College, Guilin, Guangxi, 541001, China; 2 Clinical medical school of Guilin Medical College, Guilin, Guangxi, 541001, China; 3 Guangxi Normal University, College of Life Science; Stem Cells and Medical Biological Technology Key Laboratory of Guangxi Colleges and Universities, Guilin, Guangxi, 541004, China

**Keywords:** gastric neoplasmas/gastric carcinoma, hypoxia inducible factor-1 alpha, n-myc downstream regulated gene 2, epithelial mesenchymal transformation, metastasis

## Abstract

**Objective:**

This study aims to investigate the differences in the expression of hypoxia-inducible factor-1α (HIF-1α), N-myc downstream-regulated gene 2 (NDRG2) and epithelial mesenchymal transition (EMT)-related proteins in normal gastric tissues, gastric cancer tissues and lymph node metastasis.

**Methods:**

Immunohistochemistry was used to detect the expression of HIF-1α, NDRG2, E-cadherin, Snail and Twist in normal gastric tissues, gastric cancer tissues and lymph node metastasis.

**Results:**

In normal gastric tissues, HIF-1α was not expressed, NDRG2 was highly expressed. There was a significant between the expression of NDRG2 and Snail, as well as of NDRG2 and Twist. In gastric cancer tissues, there was no statistically difference between the expression of HIF-1α and E-cadherin, NDRG2 and E-cadherin. However, there was a significant difference in expression between the expression of HIF-1α and Snail, HIF-1α and Twist, NDRG2 and Snail, and NDRG2 and Twist. In lymph node metastasis tissues, we show that HIF-1α was highly expressed, while NDRG2 was not, and the difference between the expression of HIF-1α and E-cadherin, HIF-1α and Snail, HIF-1α and Twist was not significant.

**Conclusion:**

HIF-1α may promote EMT, possibly by inhibiting the expression of NDRG2.

## 1 Introduction

The incidence of gastric cancer ranks fourth in the world, and 70% of these disease cases occur in developing countries. For example, in Asia, the incidence of gastric cancer ranks third [1] and in China, it is the third most deadly cancer following lung and liver cancer [2]. Even after R0 resection, the 1-, 2- and 3-year overall survival rates were only 89%, 74% and 63%, respectively [3], with death due to metastasis. Thus, it is critical to elucidate the mechanism of metastasis. Some studies have concluded that hypoxia-inducible factor-1α (HIF-1α) may play an important role in tumor invasion and metastasis [4, 5, 6]. Previous studies have shown that that N-myc downstream-regulated gene 2 (NDRG2) may be a cancer suppressor gene that plays a role in inhibiting the occurrence and development of tumors [7, 8, 9]. At present, the correlation between the expression of HIF-1α and NDRG2 in gastric cancer and lymph node metastasis tissues has not been reported. A key step in tumor metastasis is Epithelial mesenchymal transition (EMT) [10]. Therefore, the question to be asked is what is the association of gastric cancer metastasis with HIF-1α and NDRG2? In this study, the expression of HIF-1α, NDRG2 and EMT-related proteins, including E-cadherin, Snail and Twist were evaluated in normal gastric tissues, gastric cancer tissues and lymph node metastasis tissues.

## 2 Materials and Methods

### 2.1 General Information

From October 2014 to June 2016, 20 patients with gastric cancer confirmed by histology were admitted to our hospital, and were enrolled in this study. There were 12 males and 8 females suffering from adenocarcinoma of gastric antrum. The ages of these patients ranged from 35 to 70 years old, with a median age of 56 years old.

**Informed consent**: Informed consent has been obtained from all individuals included in this study.

**Ethical approval**: The research related to human use has been complied with all the relevant national regulations, institutional policies and in accordance the tenets of the Helsinki Declaration, and has been approved by the Ethics Committee of the Affiliated Hospital of Guilin Medical College.

### 2.2 Experimental methods

The expression of HIF-1α, NDRG2, E-cadherin, Snail and Twist in gastric cancer tissues, lymph node metastasis tissues and normal gastric tissues were identified by immunohistochemistry.

#### 2.2.1 Immunohistochemistry

The paraffin-embedded tissue sections were dewaxed, repaired, incubated with the primary and secondary antibody, colored with diaminobenzidine (DAB), re-stained with hematoxylin, and sealed. These were evaluated according to the evaluation criteria of each index.

#### 2.2.2 Evaluation criteria for HIF-1α test results [11]

HIF-1α protein is mainly expressed in the cytoplasm and/or nuclei of gastric cancer cells. The positive results were determined by a semi-quantitative scoring system: (1) Scoring was performed according to staining intensity: no color was scored as 0, light yellow was scored as 1, brownish yellow was scored as 2, and tan was scored as 3. (2) Ten high-power fields (HPF) were randomly selected from each section, and 100 epithelial cells in the gastric gland were counted from each HPF. The percentage of positive cells was evaluated as follows: no positive cells, 0 point; <25% were positive cells, 1 point; positive cells were within 25-50%, 2 points; >50% were positive cells, 3 points. These two scores were multiplied together, and results were as follows: <2 points was negative, and >2 points was positive.

#### 2.2.3 Evaluation criteria for NDRG2 results [12]

Ten HPFs were randomly selected from each section. The final score for antigen staining combined two indexes: staining intensity and stained cell proportion. The staining intensity of cells was classified into four levels: non-staining, light yellow, brownish yellow, and tan. These were denoted as 0, 1, 2 and 3 points respectively. The proportion of stained cells to the cell count was classified in five levels: 0 point, stained cells accounted for <5%; 1 point, stained cells accounted for ≥5% and <25%, 2 points, stained cells accounted for ≥25% and <50%; 3 points, stained cells accounted for ≥50% and <75%; 4 points, stained cells accounted for ≥75%. The positive intensity score of cells: Based on the staining intensity × stained cell proportion score, where (-) was 0 points, (+) was 1-4 points, (++) was 5-8 points, and (+++) was 9-12 points.

#### 2.2.4 Evaluation criteria for E-cadherin, Snail and Twist results [13]

E-cadherin is expressed in the cell membrane or cytoplasm, and yellow-stained cells are considered positive cells. Twist protein is expressed in the cytoplasm, with low expression in the nucleus. Clearly brown or tan-yellow-stained cells were considered positive cells. Ten HPFs were randomly selected from each section, and the results were determined based on the percentage of positive cells. E-cadherin: when the percentage of positive cells was ≥25%, it was considered positive; Twist: when the percentage of positive cells was ≥5%, it was considered positive; Snail: when the nucleus or/and cytoplasm presented with a light-yellow to brownish-yellow color in the staining, these were considered positive cells. The semi-quantitative analysis of cells was performed according to the staining intensity of positive cells combined with the percentage of positive cells. Staining intensity was scored based on the staining characteristics of most cells. Scoring based on comparison with the background hue: 0 point, cells are not stained; 1 point, weak positive; 2 points, positive; 3 points, strong positive. Scoring based on the percentage of stained cells: 1 point, 1-25%; 2 points, 26-50%; 3 points, 51-75%; 4 points, 76-100%. Then, the sum was calculated based on the positive cell × staining density score: a sum of 1-2 was negative, and a sum of 3-12 was positive.

### 2.3 Statistical analysis

Data was analyzed using statistical software SPSS 19.0. Count data were compared between two groups using *X2*-test. *P*<0.05 was considered statistically significant.

## 3 Results

### 3.1 Expression of HIF-1α, NDRG2, E-cadherin, Snail and Twist in different tissues

**Figure 1:**
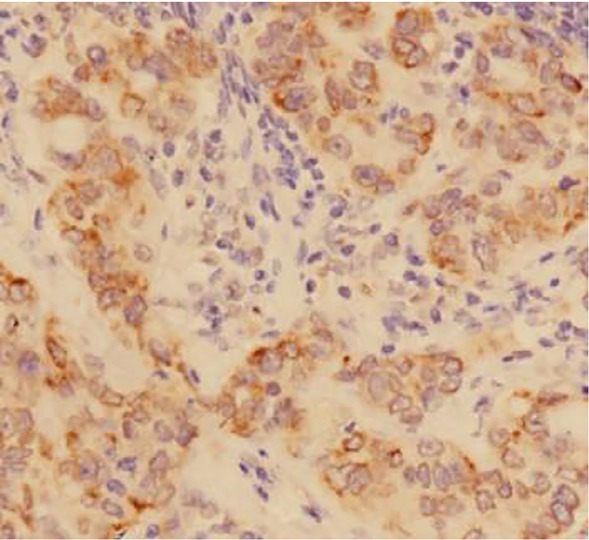
Positive expression of HIF-1 alpha in gastric carcinoma tissues (DAB staining,×400)

**Figure 2:**
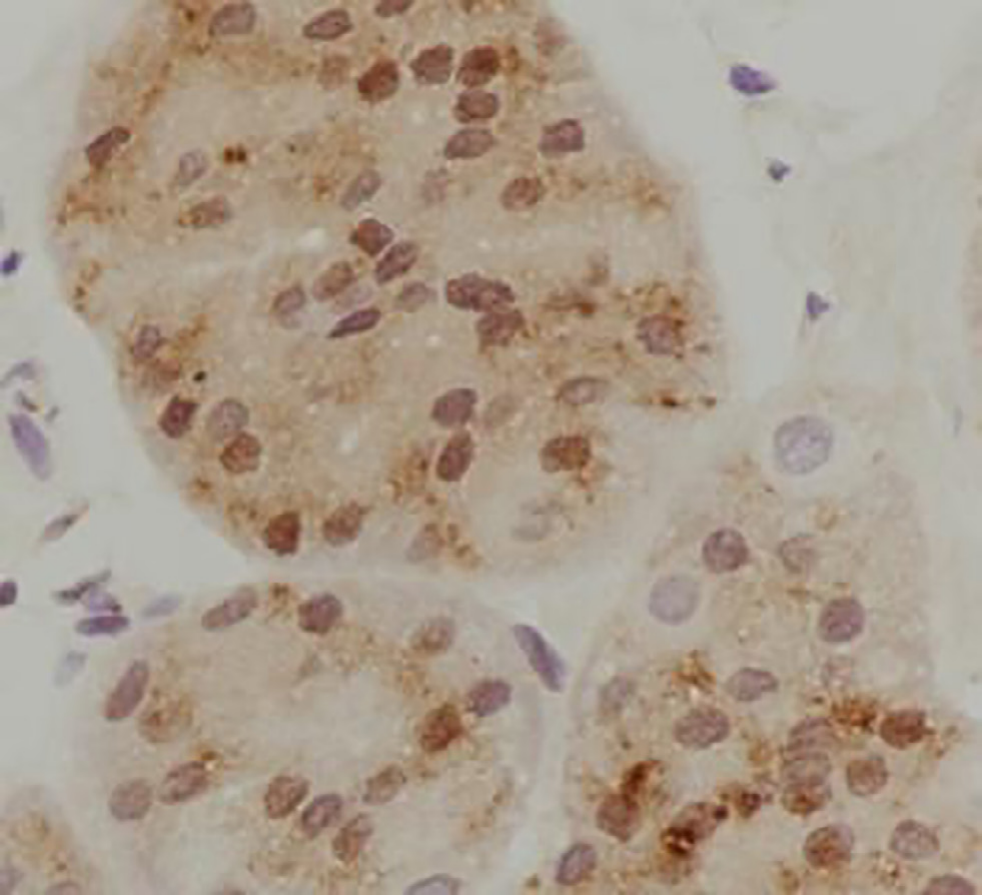
Positive expression of NDRG2 in gastric carcinoma tissues (DAB staining,×400)

**Figure 3:**
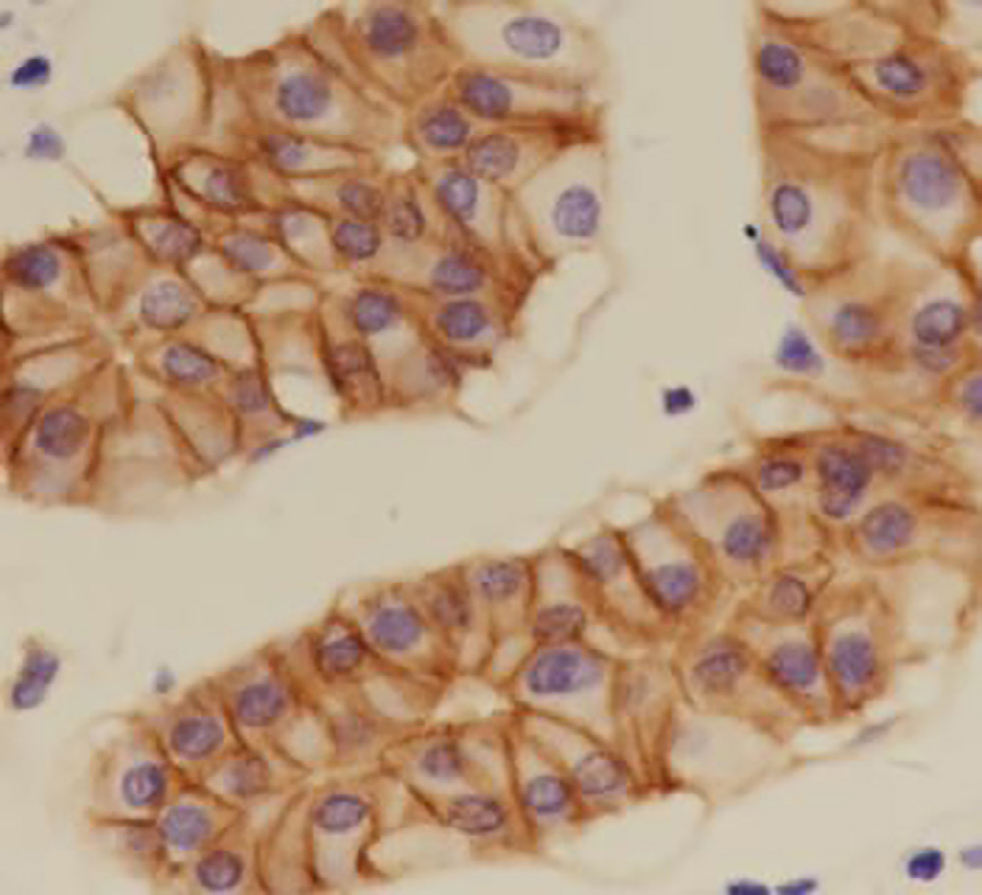
Positive expression of E-Cadherin in gastric carcinoma tissues (DAB staining,×400)

**Figure 4:**
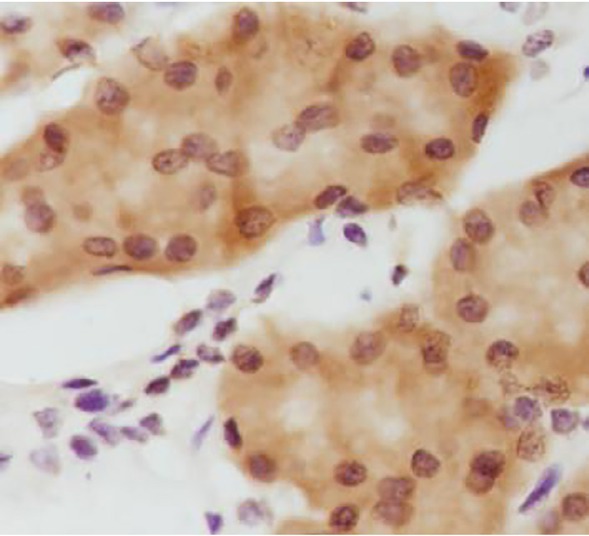
Positive expression of Snail in gastric carcinoma tissues (DAB staining,×400)

**Figure 5:**
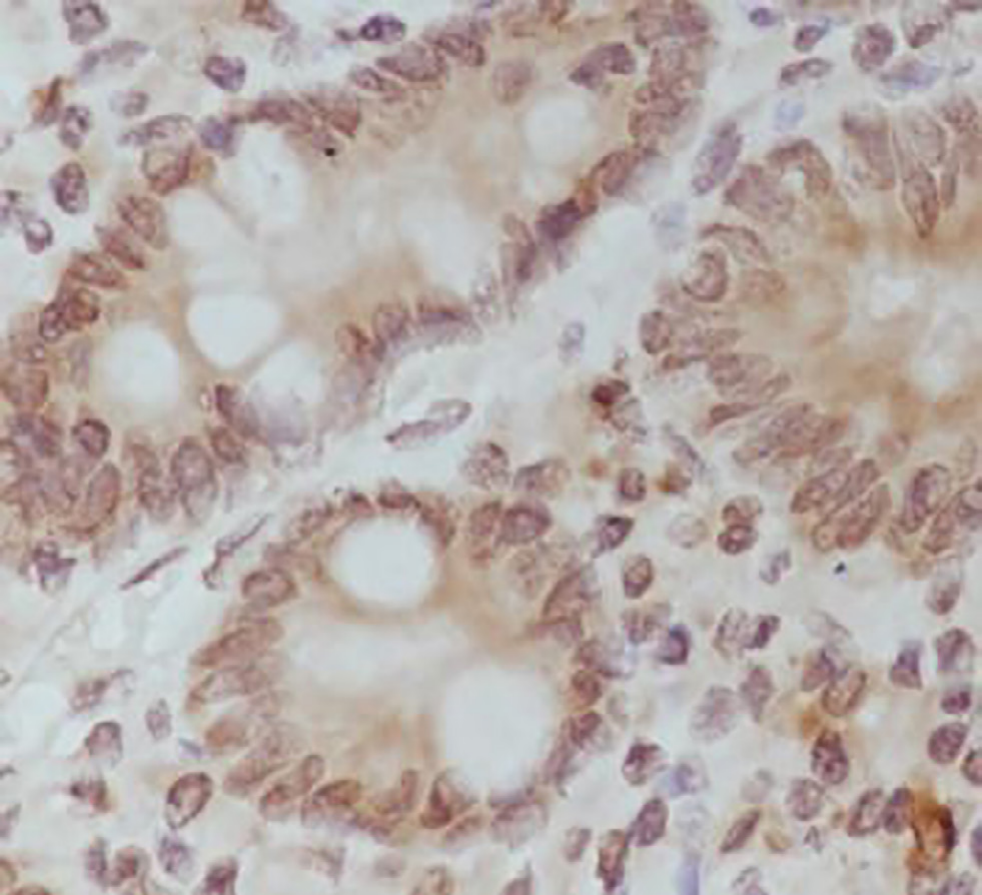
Positive expression of Twist in gastric carcinoma tissues (DAB staining,×400)

The expression results are shown in Table 1, and in Figures 1, 2, 3, 4 and 5.

**Table 1:** Expression of HIF-1 alpha, NDRG2, E-Cadherin, Snail and Twist in different tissues

	Gastric carcinoma tissue(cases)	positive rate	Lymph node tissue of metastatic carcinoma(cases)	positive rate	Normal gastric tissue(cases)	positive rate
HIF-1α	4/17	23.5%	11/19	57.9%	0/17	0.0%
NDRG2	5/20	25.0%	0/20	0.0%	17/20	85.0%
E-Cadherin	7/16	43.8%	12/18	66.7%	20/20	100.0%
Snail	12/16	75.0%	11/20	55.0%	6/17	35.3%
Twist	12/16	75.0%	13/20	65.0%	2/17	11.8%

(1) In normal gastric tissues, HIF-1α was not expressed. However, there was expression in gastric cancer tissues, and was highly expressed in lymph node metastasis tissues. There was a was statistically significant (*X2*=4.395, *P*=0.037) difference in HIF-1α expression between lymph node metastasis tissues and gastric cancer tissues. (2) NDRG2 was highly expressed in normal gastric tissues, its positive rate was up to 85%, while its expression in gastric cancer tissues was significantly decreased. There was no expression in lymph node metastasis tissues. The differences in the expression of NDRG2 among normal gastric tissues, gastric cancer tissues, and lymph node metastasis tissues were statistically significant (*X2*=30.179, *P*<0.001). There was a significant difference in expression of NDRG2 between gastric cancer tissues and lymph node metastasis tissues (Fisher, *P*=0.047). (3) The differences in the expression of E-cadherin among normal gastric tissues, gastric cancer tissues, and lymph node metastasis tissues were statistically significant (*X2*=12.217, *P*=0.000), while the differences in the expression of Snail were not statistically significant (*X2*=3.811, *P*=0.051). However, the difference in the expression of Twist was statistically significant (*X2*=15.372, *P*=0.000). Furthermore, the differences in the expression of E-cadherin, Snail and Twist between gastric cancer tissues and lymph node metastasis were not statistically significant (the *X2* values were 1.804, 1.541 and 0.419, respectively; and the *P* values were 0.179, 0.214 and 0.517, respectively).

### 3.2 Correlations among HIF-1α, NDRG2, E-cadherin, Snail and Twist in the same tissue

As shown in Tables 2, 3 and 4. In normal gastric tissues, HIF-1α was not expressed, but NDRG2 was highly expressed. The difference between NDRG2 and E-cadherin expression was not statistically significant. However there was a statistically significant difference im expression between NDRG2 and Snail, and NDRG2 and Twist.

**Table 2:** The expression of NDRG2, E-Cadherin, Snail and Twist in normal gastric tissues

	E-Cadherin		Snail		Twist	
	*x*^2^	*P*	*x*^2^	*P*	*x*^2^	*P*
NDRG2 (17/20)	Fisher	0.231	9.653	0.002	19.729	0.000

In normal gastric tissues, HIF-1 alpha is not expressed, NDRG2 is highly expressed, and there is no significant difference between E-Cadherin. There is a significant difference between Snail and Twist expression.

**Table 3:** The expression between HIF-1 alpha, NDRG2, E-Cadherin, Snail and Twist in gastric carcinoma

	E-Cadherin (7/16)		Snail (12/16)		Twist (12/16)	
	*x*^2^	*P*	*x*^2^	*P*	*x*^2^	*P*
HIF-1α	1.517	0.218	8.742	0.003	8.742	0.003
(4/17)						
NDRG2	1.406	0.236	8.916	0.003	8.916	0.003
(5/20)						

There was no significant difference between HIF-1 alpha and NDRG2 expression in gastric cancer tissues, Fisher P=1.000. The expressions of HIF-1 and NDRG2 were not significantly different from those of E-Cadherin and were significantly different from those of Snail and Twist.

**Table 4:** The expression between HIF-1 alpha, E-Cadherin, Snail and Twist in lymph node metastasis

	E-Cadherin (12/18)		Snail (11/20)		Twist (13/20)	
	*x*^2^	*P*	*x*^2^	*P*	*x*^2^	*P*
HIF-1α	0.302	0.582	0.033	0.855	0.208	0.648
(11/19)						

In lymph node metastasis tissues, HIF-1 was highly expressed and NDRG2 was not expressed. There was no significant difference between the expression of HIF-1 and the expression of E-Cadherin, Snail and Twist.

In gastric cancer tissues, the difference in the expression between HIF-1α and NDRG2 was not statistically significant, (Fisher, *P*=1.000). Furthermore, the difference in the expression between HIF-1α and E-cadherin, and the expression between NDRG2 and E-cadherin, was not statistically significant. This is in contrast between HIF-1α and Snail, HIF-1α and Twist, NDRG2 and Snail, and NDRG2 and Twist which were statistically significant.

In lymph node metastasis tissues, HIF-1α was highly expressed, while NDRG2 was not. We found that there was no statistically significant difference in the expression between HIF-1α and E-cadherin, Snail, and Twist.

## 4 Discussion

### 4.1 HIF-lα

HIF-1 is an important nuclear transcription regulating factor widely present in cells of mammals and humans under a hypoxia state. It plays an important role in the regulation of genes associated with angiogenesis, cell proliferation, energy metabolism, erythrocyte production and cell adhesion. HIF-1 is a heterodimer that consists of HIF-1α and HIF-1β subunits. Tumor invasion and metastasis is mediated by hypoxia through inducing the expression of genes via HIF-lα. Furthermore, hypoxia can induce global changes in a complex regulatory network of transcription factors and signaling proteins to coordinate cellular adaptations in metabolism, proliferation, DNA repair, and apoptosis. The accumulation of intracellular HIF-1α is due to cells under hypoxic or low glucose conditions. However, when conditions improve, this results in HIF-1α being rapidly degraded. When tumor tissues grow, they will almost certainly be in hypoxic conditions, which subsequently results in increased HIF-1α expression. Thus, the activation of oncogenes and the inactivation of cancer suppressor genes can increase the expression of HIF-1α and enhance its activity in tumor cells.

### 4.2 NDRG2

NDRG2 was first discovered by Jian Li *et al*. [13] and NDRG2 has low expression in many tumors. This includes glioma, breast cancer, colorectal cancer, gastric cancer, liver cancer and prostate cancer [14,15]. In contrast, it’s expression in normal gastric tissues is quite high. Silencing NDRG2 expression in gastric cancer cell line SNU-620 has been shown to promote the proliferation of gastric cancer cells [16]. This suggests that NDRG2 can inhibit the proliferation of gastric cancer cells.

### 4.3 EMT

EMT is mainly characterized by cell adhesion molecules. This transition results in a decrease in E-cadherin expression, and cytokeratin. On the other hand, mesenchymal marker vimentin is upregulated. Transcription factors can also be used as a biomarker for EMT, as there is an upregulation of Snail expression. Likewise, Twist inhibits E-cadherin expression and upregulate fibronectin and N-cadherin, which is characteristic of EMT. These changes are critical in tumor transformation and plays roles in tumor migration and invasion

### 4.4 Expression of HIF-1α, NDRG2, E-cadherin, Snail and Twist in different tissues

Our study shows varying levels of expression of HIF-1α and NDRG2 in different tissues. In normal gastric tissues, there was no expression of HIF-1α while expression of NDRG2 was high. In gastric cancer tissues we found that there was no difference in the expression between HIF-1α and NDRG2. When examining the expression in lymph node metastasis tissues, we found that HIF-1α was highly expressed while NDRG2 was not. These results suggests that HIF-1α may promote the development and metastasis of gastric cancer through inhibiting the expression of NDRG2. Others have previously used antibodies to HIF-1α to detect the expression of HIF-1α in various tumors. They showed that HIF-1α was highly expressed in 90% of colon, breast, and prostate cancer tissues compared to normal tissue [17, 18, 19]. E-cadherin plays a role in maintaining tight intercellular junctions, which prevents cell migration, invasion and metastasis. It remains to be elucidated whether NDRG2 is regulated by E-cadherin. In the above tissues, there was a difference between the expression of E-cadherin and Twist. However, there was no significant difference in expression between Twist and E-cadherin. The difference in the expression between Snail and E-cadherin expression was not statistically significant. This may be due to Twist inhibiting the expression of E-cadherin to promote EMT. It has been shown that the EMT of gastrointestinal cancer could be negatively regulated by the STAT3 pathway. This is through the inhibition of the NDRG2/gp130/STAT3 pathway via NDRG2 and subsequently the inhibition of tumor metastasis [20, 21, 22]. However, our results showed that there was no difference in the expression between NDRG2 and Snail. The cause of this remains to be elucidated.

### 4.5 Expression of HIF-1α, NDRG2, E-cadherin, Snail and Twist in the same tissue

Previous studies have shown that HIF-1α is expressed in various tumor tissues from liver, breast and prostate cancers [23, 24]. A number of signaling pathways, including TGF-β, PI3K, MAPK, Hedgehog and Wnt pathways are implicated in EMT. These pathways are regulated through DNA methylation, histone modification, or changes in small RNAs [25]. The transcription factor, Grhl2, can inhibit TGF-β-induced EMT. This inhibition reduces the invasion and migration ability of gastric cancer cells [26]. Likewise the inhibition of Snail can inhibit HIF-1α-induced EMT [27, 28]. In the current study, in normal gastric tissues, HIF-1α was found to not be expressed while NDRG2 was highly expressed. This is in contrast in the expression between NDRG2 and Snail, and between NDRG2 and Twist. In gastric cancer tissues, there was a statistically significant different between the expression of HIF-1α and Snail, HIF-1α and Twist, NDRG2 and Snail, and NDRG2 and Twist. In lymph node metastasis tissues, we that there was high expression of HIF-1α and no expression of NDRG2. There were no difference in the expression between HIF-1α and E-cadherin, Snail, and Twist. These results suggests that NDRG2 is closely associated to E-cadherin, Snail and Twist, while HIF-1α is not. We believe that this is due to HIF-1α affecting EMT via NDRG2.

In summary, we show that in normal gastric tissues, HIF-1α is not expressed while NDRG2 is highly expressed. This is in contrast in lymph node metastasis tissues where HIF-1α is highly expressed and NDRG2 is not. We show that the expression of NDRG2 is closely associated to the expression of E-cadherin, Snail and Twist, while HIF-1α was not. Thus, HIF-1α may affect EMT potentially by inhibiting the expression of NDRG2.
